# Evidence of Two Novel *LAMA2* Variants in a Patient With Muscular Dystrophy: Facing the Challenges of a Certain Diagnosis

**DOI:** 10.3389/fneur.2022.893605

**Published:** 2022-07-19

**Authors:** Stefanie Meyer, Silke Kaulfuß, Sabrina Zechel, Karsten Kummer, Ali Seif Amir Hosseini, Marielle Sophie Ernst, Jens Schmidt, Silke Pauli, Jana Zschüntzsch

**Affiliations:** ^1^Department of Neurology, University Medical Center Göttingen, Göttingen, Germany; ^2^Department of Human Genetics, University Medical Center Göttingen, Göttingen, Germany; ^3^Department of Neuropathology, University Medical Center Göttingen, Göttingen, Germany; ^4^Department of Diagnostic and Interventional Radiology, University Medical Center Göttingen, Göttingen, Germany; ^5^Department of Neuroradiology, University Medical Center Göttingen, Göttingen, Germany; ^6^Department of Neurology and Pain Treatment, Immanuel Klinik Rüdersdorf, University Hospital of the Brandenburg Medical School Theodor Fontane, Rüdersdorf bei Berlin, Germany; ^7^Faculty of Health Sciences Brandenburg, Brandenburg Medical School Theodor Fontane, Rüdersdorf bei Berlin, Germany

**Keywords:** muscular dystrophy, LGMDR23, hereditary myopathy, merosin, next generation sequencing

## Abstract

**Background:**

Benefits and challenges resulting from advances in genetic diagnostics are two sides of the same coin. Facilitation of a correct and timely diagnosis is paralleled by challenges in interpretation of variants of unknown significance (VUS). Focusing on an individual VUS-re-classification pipeline, this study offers a diagnostic approach for clinically suspected hereditary muscular dystrophy by combining the expertise of an interdisciplinary team.

**Methods:**

In a multi-step approach, a thorough phenotype assessment including clinical examination, laboratory work, muscle MRI and histopathological evaluation of muscle was performed in combination with advanced Next Generation Sequencing (NGS). Different in-silico tools and prediction programs like Alamut, SIFT, Polyphen, MutationTaster and M-Cap as well as 3D- modeling of protein structure and RNA-sequencing were employed to determine clinical significance of the *LAMA2* variants.

**Results:**

Two previously unknown sequence alterations in *LAMA2* were detected, a missense variant was classified initially according to ACMG guidelines as a VUS (class 3) whereas a second splice site variant was deemed as likely pathogenic (class 4). Pathogenicity of the splice site variant was confirmed by mRNA sequencing and nonsense mediated decay (NMD) was detected. Combination of the detected variants could be associated to the LGMDR23-phenotype based on the MRI matching and literature research.

**Discussion:**

Two novel variants in *LAMA2* associated with LGMDR23-phenotype are described. This study illustrates challenges of the genetic findings due to their VUS classification and elucidates how individualized diagnostic procedure has contributed to the accurate diagnosis in the spectrum of LGMD.

## Introduction

*LAMA2*-associated muscular dystrophies (LAMA2 MD) are autosomal recessive diseases caused by variants in the *LAMA2* gene, which encodes the Laminin-α2 chain of Laminin-2 (1). Laminin-2 (synonym: merosin) is the isoform of laminin in the basal membrane of skeletal muscle (2). It is cleaved into a 300 kDa N-terminal and an 80 kDa C-terminal segment (3). The N-terminal domain nucleates the association to the basal lamina of the muscle as well as to other laminin chains whereas the C-terminal domain is needed for receptor binding to integrin-α7β1 and the dystroglycan protein complex (4).

The first mutation in *LAMA2*, causing a truncated protein with loss of function and reduced muscle stability, was described in 1994 (5). The range of different clinical phenotypes associated with LAMA2 MD has increased during the last years through advances in molecular genetic analysis (6). A deficiency in the expression of laminin-2 is often observed in patients with congenital muscular dystrophy (CMD), which is characterized by severe hypotonia, the inability to walk and delayed motor development (7, 8).

Pathogenic missense variants, in-frame deletions and splice variants can lead to the more benign form, the late-onset limb girdle muscle dystrophy (LGMDR23) while CMD cases are more frequently associated with non-sense or out-of-frame mutations. Patients with LGMDR23 show delayed motor development but gain the ability to walk. They often develop scoliosis, a rigid spine, joint contractures and respiratory difficulties (9, 10). Muscle MRI typically shows abnormal muscle fat replacement in a distinct pattern. The muscles predominantly involved include subscapularis, lumbar paraspinal muscles, gluteus minimus and medius, posterior thigh and soleus muscles, whereas a sparing of levator scapulae, the forearm, the anterior leg muscles, gracilis, sartorius and adductor longus muscles is characteristic (11). Brain MRI shows confluent hyperintense lesions predominantly in the periventricular white matter with involvement of the subcortical U fibers. The corpus callosum is typically spared and no ventricular dilation is present (12).

## Materials and Methods

Clinical examination, laboratory work and imaging procedures were part of the basic evaluation regarding the patients' symptoms and performed by trained personnel according to standard protocols. All data was obtained and published with the written, informed consent of our patient. Muscle biopsy of the right biceps femoris was performed by a trained neurosurgeon under local anesthesia. Biopsied muscle was chosen regarding clinical presentation and MRI results. Biopsy tissue was snapfrozen in −80°C isopentane, cryosectioned at 6 μm and air dried for 10 min. After blocking in 3% H_2_O_2_ for 10 min, sections were stained against merosin (Novocastra, 3/22B2, mouse, 1:100 in PBS), and finally developed using DAKO Envision Peroxidase/DAB detection system (rabbit/mouse).

For NGS, genomic DNA was isolated from a blood sample and subjected to a diagnostic panel (TruSight One panel, 4,813 genes), using the HiSeq 2500 sequencing platform from Illumina. The identified variants were confirmed by Sanger-Sequencing. Detected variants were classified according to ACMG guidelines. 3D modeling of the protein structure was visualized using UCSF Chimera software (13). In order to identify effects of variants in protein sequence on the molecular structure, the Dunbrack Rotamer library 2010 was used inside Chimera (14). For analysis of splice site effects of the detected variants in *LAMA2*, RNA was extracted from PaxGene blood samples (control and patient). Using primers located in exon 55 and 58 (5'- GGTCAGCTCTCCCAGCGATTCTCCCCTGTGCCAATTGAT−3' and 5'-CAGCTCTCCCACAGGCGAAATGTG TCAGAACTGGGGTGG−3'; Eurofins) of the *LAMA2* gene, an amplicon was created and separated on an agarose gel for visualization. The sequencing reaction was performed using BigDye and Universal Adapter Primers (Eurofins) and sequencing was performed on an ABI3500 sequencer (ThermoFisher). The patients' result was compared to two samples from healthy controls. Quantitative RT-PCR was performed using SYBR Green (QIAGEN) on a QuantStudio 5 (ThermoFisher) in three biological replicates with two different primer pairs for *LAMA2* (q1F: 5'- GGCCGGTACTGTGAGCTCTGTG-3'; q1R: 5'- GACCCTGAACGTTGGCTCTGC-3'; q3F: 5'-CCCAAAGCCCAAACCCAGCATC-3'; q3R: 5'-GCCTGTGCCTTTGGTGAGCTTC-3'; Eurofins) and two housekeeping genes *PPIA* (F:5'-GACTGAGTGGTTGGATGGCA-3' and R:5'-TCGAGTTGTCCACAGTCAGC-3'; Eurofins) and *GAPDH* (F:5'-catcaccatcttccaggagc-3'and R:5'-atgaccttgcccacagcctt-3'; Eurofins) in duplicates. Relative *LAMA2* expression was calculated using ddCT method and a paired *t*-test was performed.

## Results

We report on a 41-year-old male, Caucasian patient from Germany with slowly progressing muscular dystrophy. His first symptoms developed at an age of 3 to 4 years with difficulties getting up from a lying position. At the age of 28, his walking ability became gradually impaired and several falls occurred. The clinical examination revealed proximal paresis of both arms and legs, weakness of thoracic muscles, bilateral scapulae alatae, a hyperlordosis of the lumbar spine and foot malalignment on both feet. There was no history of neuromuscular disease in the patients' family.

Creatine kinase (CK) increase by 4-fold above the upper reference level was observed. The electromyogram displayed myopathic changes. A whole-body MRI showed muscle atrophy and conversion of muscle into fatty tissue, in a pattern compatible with LGMDR23 (11). In muscle biopsy, typical myopathic impairment, including internalized nuclei and variation of the fiber size, was apparent. Extensive immunohistochemical staining, especially focusing on LGMD, had failed to produce a reliable diagnosis. Interestingly, staining of merosin, using an antibody binding the 300 kDa merosin-fragment, appeared normal (data not shown).

We suspected a hereditary muscular dystrophy. Since previous molecular genetic testing (LGMD panel diagnostics containing 10 genes) did not offer any pathologic results, we performed Next Generation Sequencing (NGS) of a panel for disease-associated genes (TruSight One, 4,813 genes). Two heterozygous variants in *LAMA2* were found. To verify a *trans* configuration of the two variants, a segregation analysis was performed. Because the patients' parents have already passed away and no other family member was affected by the disease, we carried out a segregation analysis on DNA samples from two healthy brothers. Only one variant could be detected, pointing to a compound heterozygous status of the variants in the patient.

**Figure 1:**
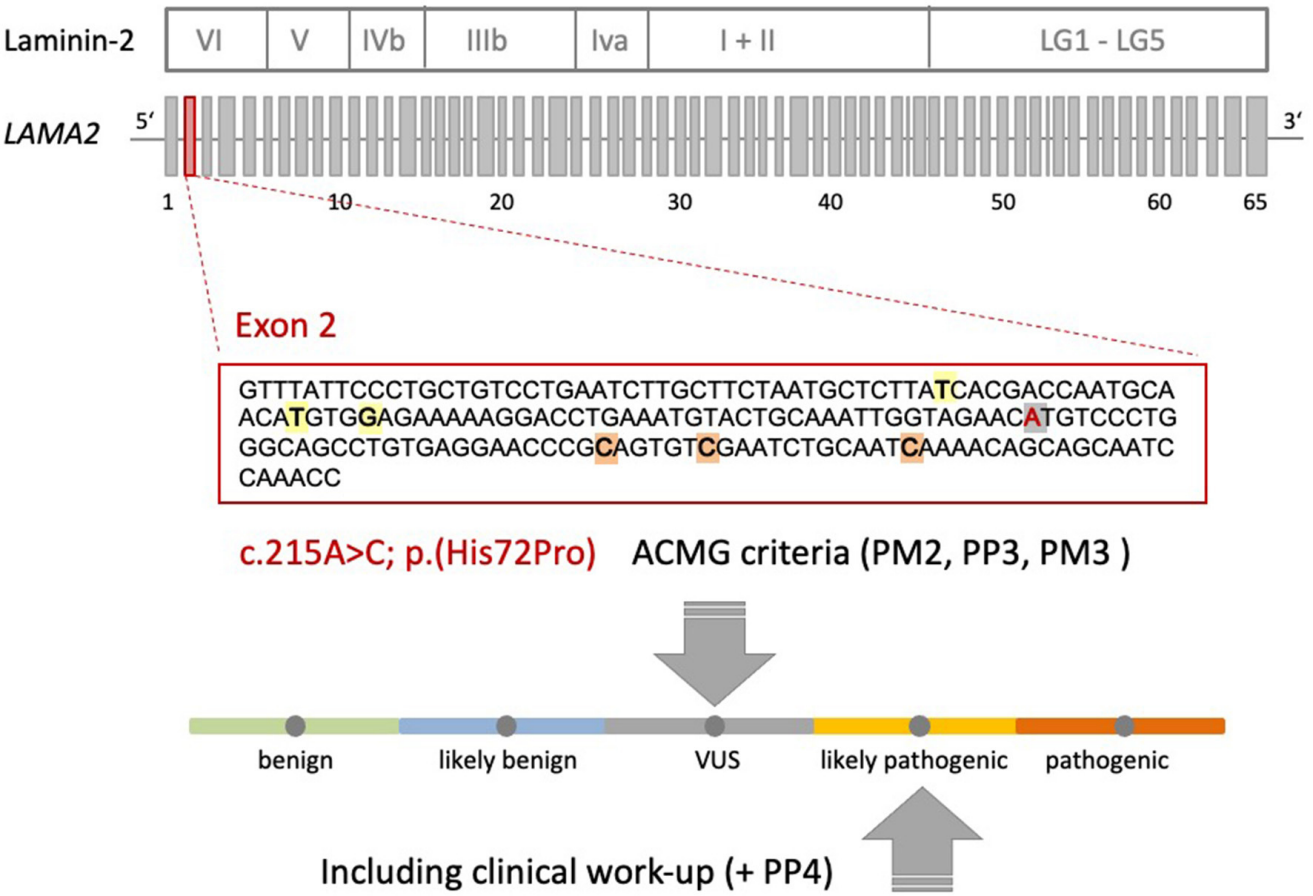
Schematic representation of the Laminin-2 protein, the gene architecture of *LAMA2* and the VUS c.215A>C (p.(His72Pro)) detected in our patient. *LAMA2* consists of 65 exons. According to HGMD® Professional 2021.4, three pathogenic non-sense variants and three missense variants with uncertain significance are described in exon 2 of *LAMA2* in the context of muscular dystrophy. Orange: non-sense variants: c.244C>T (p.Q82*), c.250C>T (p.R84*), c.283C>T (p.Q95*); yellow: missense variants: c.155T>A (p.I52N), c.172T>C (p.C58R), c.176G>A (p.G59E); gray: VUS c.215A>C (p.His72Pro) detected in our patient. The ACMG criteria leading to the initial classification “VUS” are shown. Additional clinical work-up (e.g., MRI scan) revealed a clinical phenotype and MRI pattern consistent with LGMDR23. Therefore, the VUS could be re-classified as likely pathogenic (+PP4).

**Figure 2:**
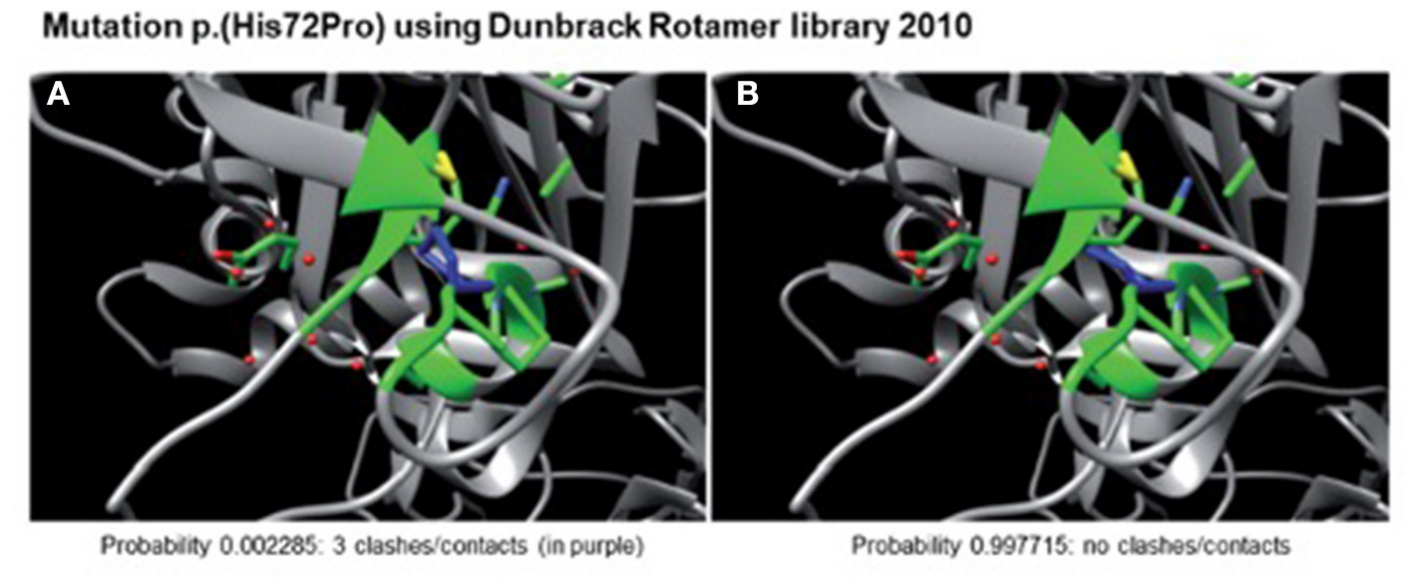
Modeled 3D-structure resulting from c.215A>C in exon 2 of LAMA2. Simulation using the Dunbrack Rotamer library 2010 shows two different 3D conformations of the mutated amino acid (in blue). All neighboring amino acids with a possible interaction with the mutated one are shown in green. Confirmation **(A)** would produce three clashes and is therefore highly unlikely to occur (calculated probability of 0.002285), whereas confirmation **(B)** produces no clashes and is therefore the preferred confirmation (probability of 0.997715) leading to no alterations in protein structure.

A heterozygote sequence alteration NM_000426.4:c.215A>C (NC_000006.11:g.129371165A>C) was detected in exon 2 of the *LAMA2* gene, which leads to the amino acid change of histidine to proline in position 72 (p.(His72Pro)). This amino acid change was predicted to be damaging per different analysis tools like POLYPHEN2, SIFT, Mutationtaster, Mutation asseccor, MutPred and M-CAP (REVEL: 0.86 deleterious). The variant was not present in gnomAD database (gnomAD v2.1.1; (15)). The CADD score at this position and variant was 26.0. According to ACMG guidelines, the variant was initially classified as a variant of uncertain significance using criteria PM2, PP3 and PM3 (detected in *trans* with a second pathogenic variant) (Figure 1) (16). The variant was submitted to ClinVar (SCV002511377). Modeling of the 3D structure of domain IV of human netrinG2, a protein crystallizing an equivalent domain to merosin with corresponding protein sequence, did not show relevant changes in the proteins‘ secondary structure resulting from this variant compared to the wild type (Figure 2). Dunbrack Rotamer library 2010 was used for 3D modeling of protein structure (14).

**Figure 3:**
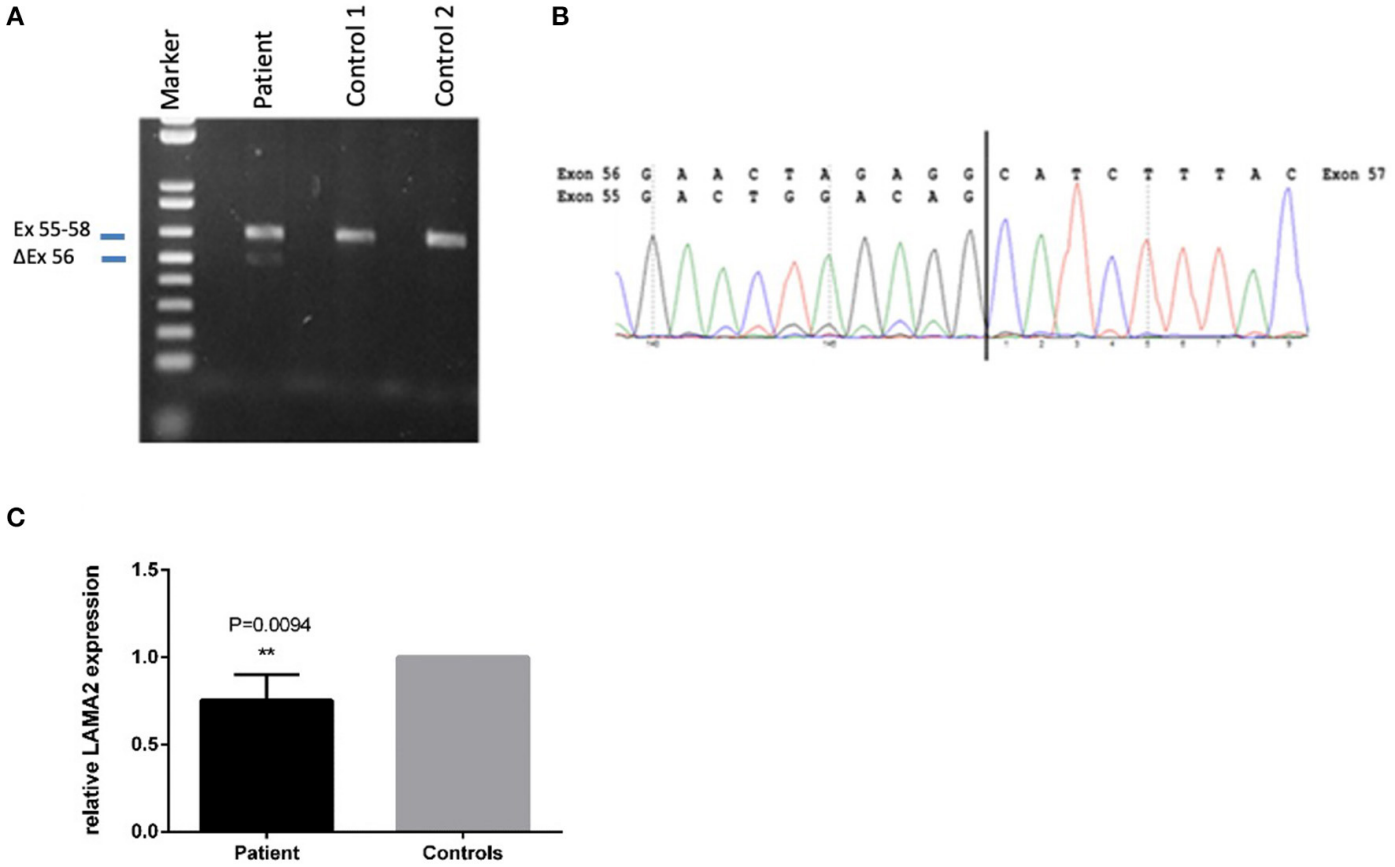
Sequencing of the LAMA2 RNA from patients‘ blood revealed partial exon-skipping of exon 56. **(A)** Proof of partial exon skipping of exon 56 on cDNA from patients' blood compared to two control samples. Upper band 629 bp (corresponding to the estimated size of exon 55–58), lower band 480 bp (corresponding to the estimated size of exon 56 skipping). **(B)** mRNA base sequence of the patient from exon 55 to 57. **(C)** Quantitative RT-PCR on patients cDNA in comparison to three wild type controls in three biological replicates. *PPIA* and *GAPDH* were used as housekeepers. The relative *LAMA2* expression was calculated using the ddCT method. A significant lower expression of the *LAMA2* transcript was observed (**p < 0.01), demonstrating a partial non-sense mediated decay (NMD).

A second heterozygote sequence alteration NM_000426.4:c.7750-2A>G in intron 55 of the *LAMA2* gene was observed (NC_000006.11:g.129807617A>G). This variant probably directly impairs the acceptor splice site as predicted by in silico analysis tools like dbscSNV Ada (deleterious (1)), RF (deleterious (0.94)) and varSEAK (class 5, splice site lost). Thus, despite a correct mRNA synthesis, loss of the exon or the use of an alternative splice position can cause severe changes in protein structure. Regarding the ACMG guidelines, it can be classified as likely pathogenic (class 4) using criteria PVS1_very strong and PM2 (16). The variant was submitted to ClinVar (ID 689499). To analyze the influence of the variant at the RNA level, we performed mRNA sequencing using a blood sample of the patient. This analysis showed partial exon skipping of exon 56 in the patients‘ sample compared to controls (Figure 3). On mRNA level the mutation results in the loss of coding position c.7750_7898 (NM_000426.4 (LAMA2_v001):c.7750_7898del) leading to premature truncation of the LAMA2 protein (p.(Ala2584Hisfs^*^8)). To analyze if the observed partial exon skipping is due to nonsense mediated mRNA decay (NMD) of the allele affected by the splice site variant, quantitative RT-PCR was performed (Figure 3). In comparison to three wild type controls, we observed a significant (*p* = 0.0094) lower presence of *LAMA2* transcripts in the patient in line with a partial NMD.

**Figure 4:**
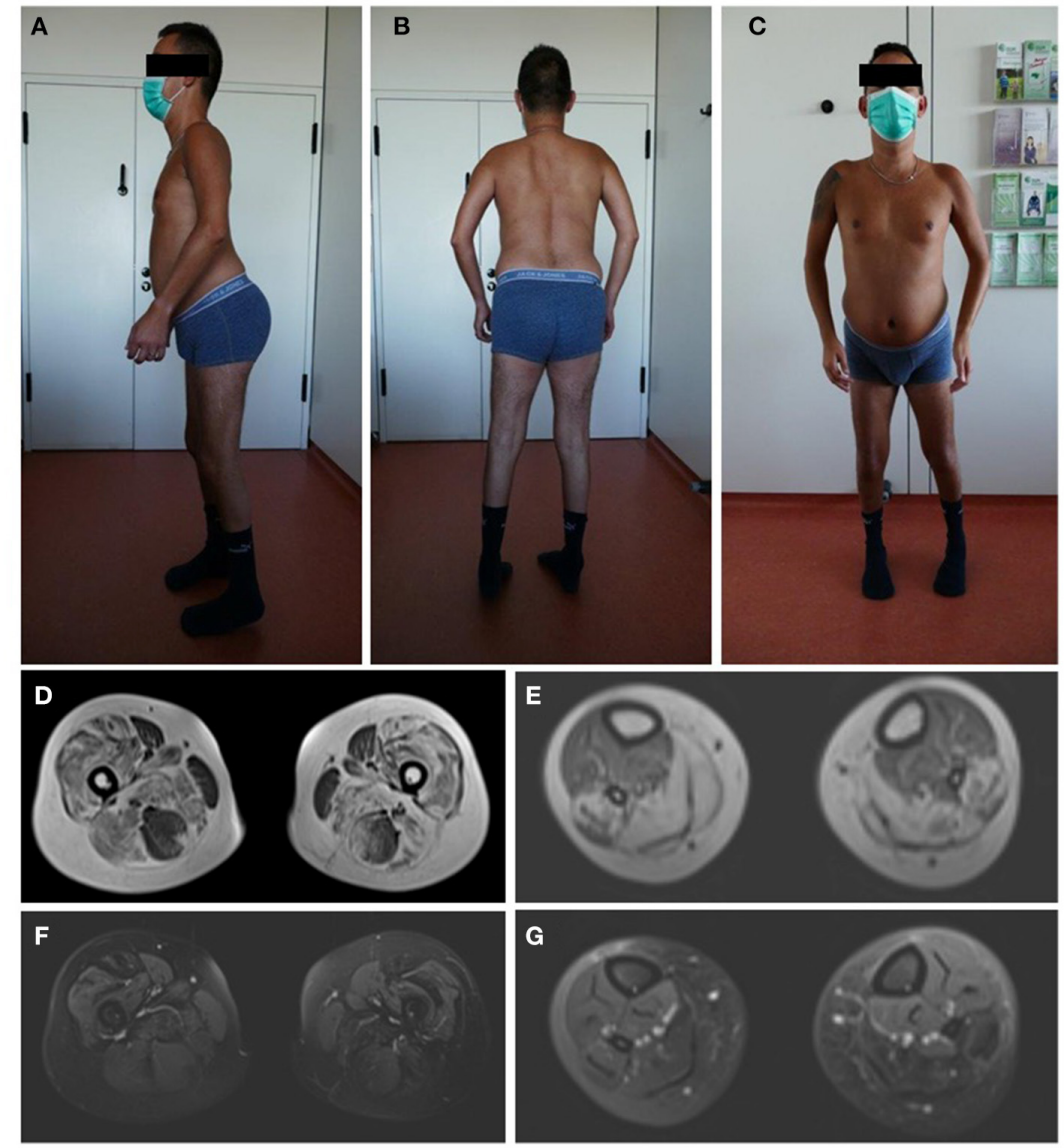
Patients‘ phenotype correlates with LGMD-specific MRI pattern. Photographs of the patient demonstrate muscle atrophy of the legs and a rigid spine syndrome with joint contractures, most prominent in the elbows and achilles tendons **(A–C)**. T1-weighted imaging in axial orientation of the thighs and calves revealed fatty infiltration and atrophy of the muscles, predominantly of the quadriceps, adductor magnus and longus, biceps femoris and soleus with sparing of the gracilis, sartorius, semimembranosus muscle and anterior compartment of the lower leg **(D,E)**. Additional proton-density-weighted imaging of the lower extremity was performed with no sign of edema in the above mentioned muscle groups as a sign for active myositis **(F,G)**.

**Figure 5:**
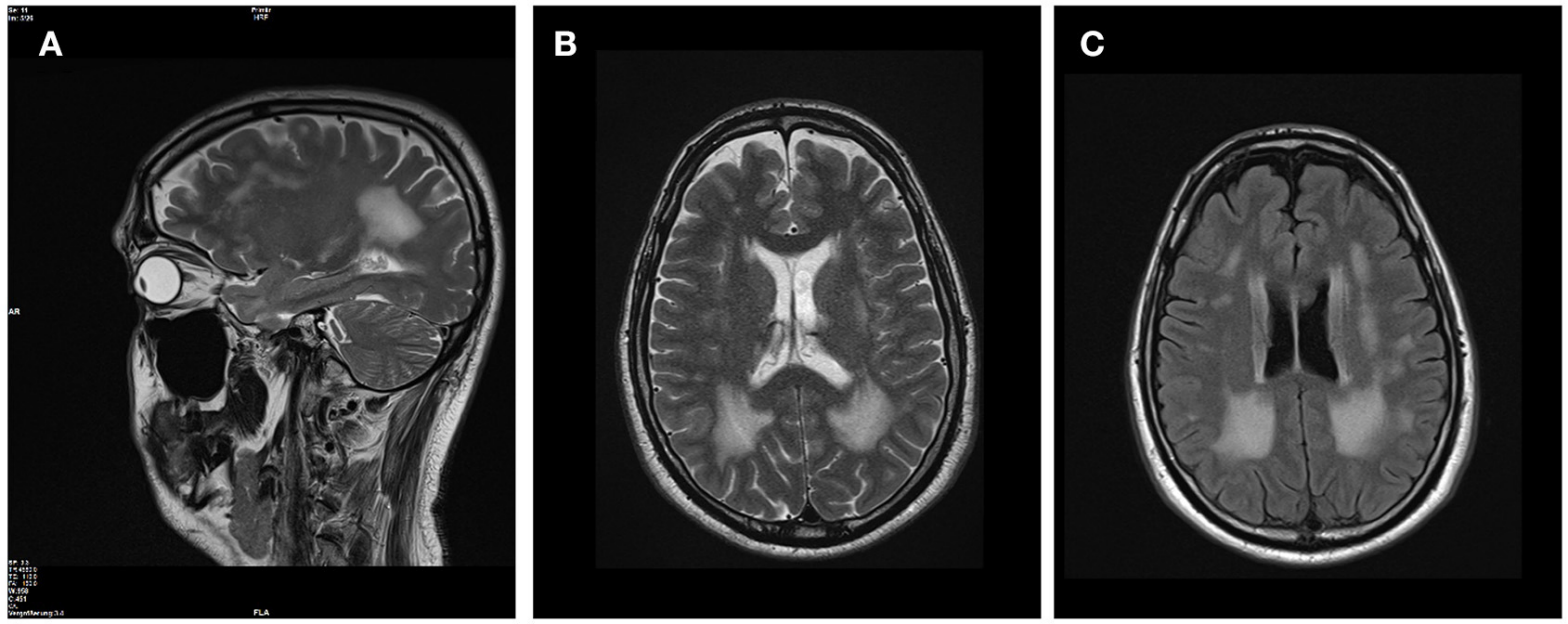
Brain MRI revealed white matter lesions typical for LAMA2-associated diseases. T2-weighted sagittal **(A)** and axial **(B)** brain MRI images revealed confluent hyperintense lesions predominantly in the periventricular white matter with involvement of the subcortical U fibers. The corpus callosum was spared, the ventricles are not enlarged and no other intracranial malformations were present **(C)** on FLAIR-weighted axial scan.

The patients' phenotype was typical for a mild to moderate LGMDR23, with a slowly progressive proximal muscular weakness beginning in early childhood with rigid spine syndrome and joint contractures most prominent in the elbows and Achilles tendon (Figure 4). In addition, the muscle MRI revealed a typical pattern of ubiquitous fat replacement. The autochthonous lumbar muscles, the quadriceps muscle and the posterior thigh muscles, predominantly adductor magnus and biceps femoris, were affected. Moreover, the typical sparing pattern of the gracilis, sartorius and semimembranosus muscles could be observed (Figure 4). Results of cranial MRI supported LGMDR23 diagnosis by displaying characteristic white matter lesions (Figure 5).

## Discussion

Two previously unknown variants in *LAMA2* (c.7750-2A>G, c.215A>C) can be associated with LGMDR23-phenotype. We detected a persistent expression of merosin in the skeletal muscle by immunohistochemistry, whilst using antibodies directed against the amino and carboxyl terminus of the protein (6), which was consistent with the milder phenotype of this patient.

Regarding the variant c.7750-2A>G, we expected skipping of exon 56, which could possibly result in loss of protein through NMD (17). RNA analysis showed partial skipping of exon 56 in the sample of our patient, and subsequent quantitative RT-PCR demonstrated a lower expression of *LAMA2* in the patients' blood, indeed pointing to a low but significant NMD in case of the splice site variant c.7750-2A>G.

Due to the low level of NMD, this reduction in gene expression might not be detectable on protein level using immunohistochemistry techniques, explaining the normal histopathological result.

**Figure 6:**
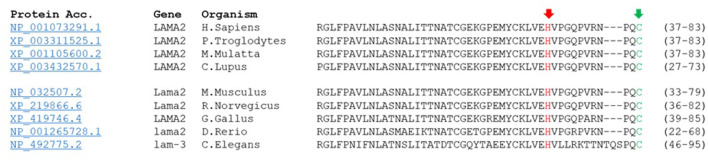
His72 is conserved in laminin α2-protein sequence. His72 is switched through missense variant c.215A>C to proline. Alignment of laminin α2 peptide sequences from human, chimpanzee, rhesus monkey, wolf, mouse, rat, chicken and fish and their homolog in C. elegans shows phylogenetic conversation of His72 at this position (red arrow). Moreover, peptide sequence reveals close proximity of the peptide switch to Cys79 (CxxC motif, green arrow).

The detected missense variant c.215A>C results in an exchange of histidine to proline in position 72. His72 is highly conserved throughout species and, therefore, can be suspected to maintain a relevant role in protein function (Figure 6).

Structural protein alterations of laminin-α2 can lead to a reduced stability of the sarcolemmal zone, a higher vulnerability against contraction-induced stress or an impairment of the proteins' ability to polymerize with the muscle fiber basement membrane and therefore result in muscle fiber damage (18). As far as the crystal structure of laminin-α2 has not yet been completely deciphered (19, 20), we used the modeling of the 3D structure of domain IV of human netrinG2. No relevant changes in the proteins‘ secondary structure resulted from this variant compared to the wild type.

According to literature and catalogued *LAMA2* variants in the *LAMA2* gene variant database (21), pathogenic missense variants seem to cluster in specific regions of laminin-α2, one cluster localized in domain IV corresponding to the N-terminal part of laminin-α2 (21, 22). Laminin N-terminal domains (LN domains) are found on other proteins of the extracellular matrix as well as netrins. They are necessary in self-assembly into high-ordered networks for laminin-polymerization and skeletal muscle stability (19, 23). The modeled 3D protein structure of domain IV did not show any relevant alterations. Various assumptions can be made for this, e.g. that there will be a conformational change of another part of the total protein which is not depicted in the 3D model, or the missense variants in this region can disturb protein self-assembly into supramolecular networks and therefore might disrupt protein function fundamentally (24, 25).

Mutations in domain IV of merosin have been shown to be associated with muscular dystrophy. In a mouse model for MDC1A, a missense mutation (p.(Cys79 Arg)) in the highly conserved CxxC motif was analyzed in detail and was found to affect the myelination of Schwann cells and the stability of muscles cells (22). Interestingly, extensive *ex vivo* testing of this variant revealed that the phenotype was not caused by reduced protein levels, protein mislocalization or changes in ECM-composition (22), which is in line with the observations, we made in our patient. The missense variant described in our case is located in very close proximity to the CxxC motif, separated by only ten amino acids (Figure 6). The CxxC motif is found in all known laminin LN domains. It is suspected to be involved in a more specific molecular interaction, elemental for laminin α2-function. Furthermore, it might be required for high-level organization of laminin meshwork within the mature basal lamina (26, 27). Overall, those previous descriptions of missense mutations in the domain IV of laminin suggest relevance of this area in protein-protein interaction, not sufficiently mirrored by protein abundance or localization, thus supporting the association of the newly detected missense variant with the LGMDR23 phenotype our patient displayed.

Previous studies demonstrated a milder phenotype in patients with residual merosin and described a genotype-phenotype correlation in *LAMA2*-related muscular dystrophies (6, 9). A recent study on a cohort of Chinese patients with *LAMA2*-related muscular dystrophy revealed an association of LGMDR23 with missense variants, leading to the hypothesis that missense pathogenic variants could be a predictor of a milder phenotype (28).

The described clinical phenotype typical for LGMDR23 and the distinct MRI pattern of muscle and brain further confirm the pathogenicity of the genetic variants. According to this, we reclassify the missense variant to a likely pathogenic variant by adding ACMG criteria PP4 to the above-mentioned criteria (Figure 1).

## Conclusions

Our findings underscore the importance of NGS in diagnostics of neuromuscular disorders. Muscle biopsies play an important role in accelerating the pathogenicity of newly detected DNA sequence variations and are highly relevant in diagnosis of genetic neuromuscular diseases but do not always lead to satisfying results. Through increasing usage of NGS, more variants of unknown significance will be detected in the future. Thus, close interdisciplinary exchange and correlation to the subjects' phenotype is extremely important to determine clinical significance of new genetic findings. For further research, it will be of imminent importance to document clinical data and *LAMA2* variants in international databases in order to accelerate the geno- and phenotypic spectrum of LGMD. Furthermore, it will be necessary to develop strategies from bedside to bench for functional analysis and validation of new variants, especially regarding predictability of missense variants. One possible solution could be *ex vivo* cell culture assays obtained from a biological sample of the individual patient. With the presented case and through the careful workup, we can contribute to the genotype-phenotypic spectrum of LGMD.

## Data Availability Statement

The datasets presented in this article are not readily available because of ethical and privacy restrictions. Requests to access the datasets should be directed to the corresponding author/s.

## Ethics Statement

Ethical review and approval was not required for the study on human participants in accordance with the local legislation and institutional requirements. The patients/participants provided their written informed consent to participate in this study. Written informed consent was obtained from the individual(s) for the publication of any potentially identifiable images or data included in this article.

## Author Contributions

Conceptualization and project administration: SM, JZ, and SP. Methodology: SZ and SK. Investigation and resources: SM, SZ, SK, SP, and JZ. Data curation: SM. Writing—original draft preparation: SM, SZ, SK, ME, and KK. Writing—review and editing: AS, JS, SP, and JZ. Visualization: SM, AS, ME, KK, SZ, SK, and SP. Supervision: SP and JZ. All authors have read and agreed to the published version of the manuscript.

## Funding

SM was supported by the Deutsche Forschungsgemeinschaft (DFG, German Research Foundation) within the Clinician Scientist Program “Cell Dynamics in Disease and Therapy” at the University Medical Center Goettingen (project number 413501650). All authors are members of the European Reference Network for Rare Neuromuscular Diseases (ERN EURO-NMD). JZ is supported by Horizon-2020: IMI2-2020-23-05 (grant number 101034427-2). We acknowledge support by the Open Access Publication Funds of the Göttingen University.

## Conflict of Interest

Unrelated to this study SM has received payments for travel expenses from Alnylam, Kedrion, Momenta Pharmaceuticals, and Biogen. JS has received payments for advisory boards, speakers honoraria, travel expenses, research projects from Alnylam, Argenx, Biogen, BioMarin, Biotest, CSL Behring, Kezar, LFB, Novartis, Octapharma, Pfizer, and UCB. JZ has received payments for advisory boards, speakers honoraria, travel expenses, research projects from Alnylam, Biogen, Biotest, CSL Behring, Octapharma, Kedrion, Grifols, UCB, Hormosan, Alexion, and Sanofi. The remaining authors declare that the research was conducted in the absence of any commercial or financial relationships that could be construed as a potential conflict of interest.

## Publisher's Note

All claims expressed in this article are solely those of the authors and do not necessarily represent those of their affiliated organizations, or those of the publisher, the editors and the reviewers. Any product that may be evaluated in this article, or claim that may be made by its manufacturer, is not guaranteed or endorsed by the publisher.
